# Supplementary figures and images for: Semaphorin 3f and post-embryonic regulation of retinal progenitors
Source: PLoS Genet. 2025 Jul 14;21(7):e1011748. doi: 10.1371/journal.pgen.1011748 (PMC12274001; doi:10.1371/journal.pgen.1011748)

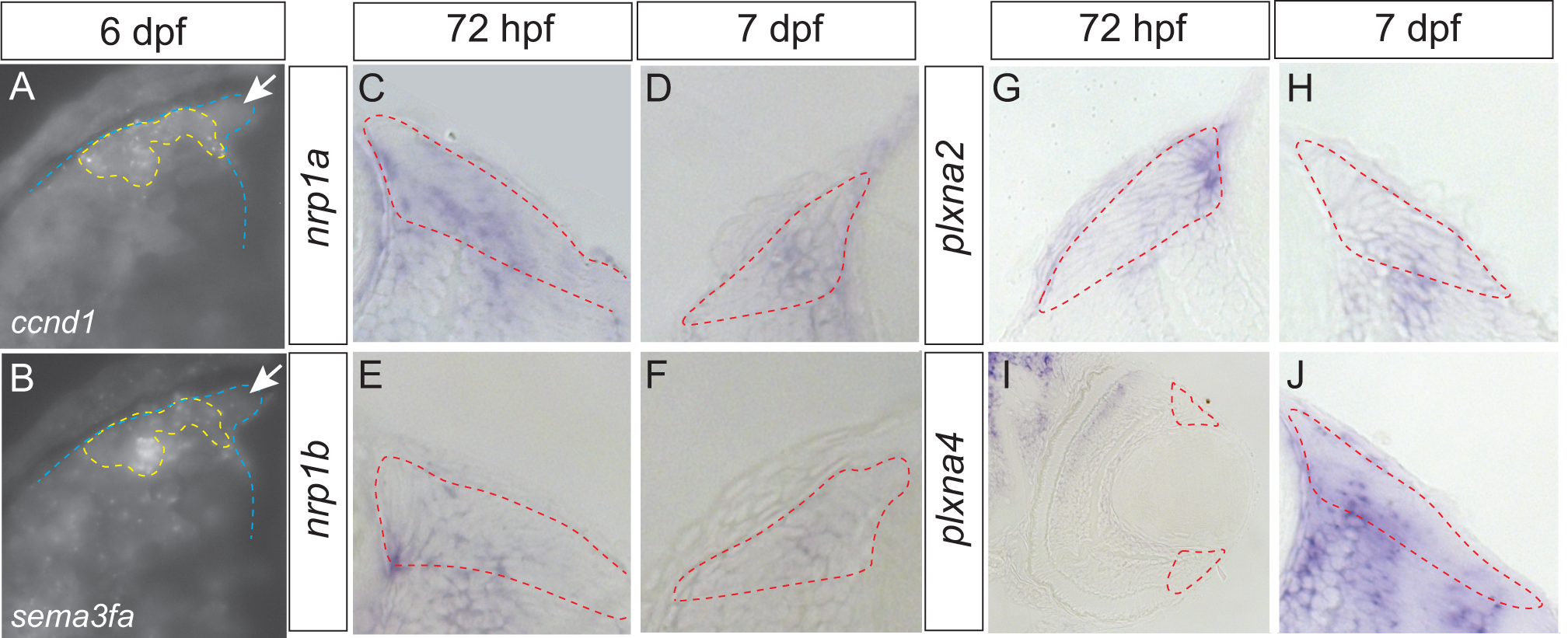

Supplement: S1 Fig — A-B) Double FISH on a cryostat retinal section for ccnd1 (A) and sema3fa (B) mRNA in the CMZ (outlined with blue dashed line), with the ccnd1 domain represented in B by a dotted yellow line. sema3fa mRNA largely overlaps with ccnd1, but neither gene is expressed in the most distal CMZ where the retinal stem cells reside (white arrows). C-J) Plastic sections of whole mount 72 hpf (C, E, G, I) and 7 dpf (D, F, H, J) larvae processed for in situ hybridization with antisense riboprobes for nrp1 (C-F) and plxna (G-J) receptors. (TIF) [file pgen.1011748.s001.tif]
