# Supplementary material for: Semaphorin 3f and post-embryonic regulation of retinal progenitors
Source: PLoS Genet. 2025 Jul 14;21(7):e1011748. doi: 10.1371/journal.pgen.1011748 (PMC12274001; doi:10.1371/journal.pgen.1011748)
Supplement: Attachment — Submitted filename: Reviewer response PLOS Genetics April 2025.pdf [file pgen.1011748.s003.pdf]

Reviewer #1:

The Reviewer felt "In general the data shown are of high quality and conclusions are in line with results." They did raise concerns about sample sizes and the negative data with the gain-of-function studies of Sema3fa. We address these concerns and others raised below.

1. Sample sizes of N=1 or 2 is no acceptable for studies in Figures 2, 4, 7.

We raise the possibility that the Reviewer misunderstood the data in our graphs in these Figures. The sample sizes are much larger than N=1 or 2. The "N" are the numbers of independent replicates, but the numbers of biological replicates (n) (i.e. individual larva) is generally >8-10. Importantly, each point in a graph represents an individual larva. We indicate as such in the Figure Legends for all our figures. Note that we have added an additional experimental replicate to the *bmp4* data in Figure 4G, and we now show correctly that the pHH3+ data in Figure 2 was pooled from N=3 independent experiments.

2. The heat shock, over-expression studies are difficult to interpret as no effect was found. Perhaps mosaicism or extent of expression was too low. A positive control or better – germ-line transgenic analysis might be needed. In general, I did not find the studies compelling in design and could simply be removed.

All three Reviewers felt that the Sema3fa overexpression data were difficult to interpret, with mosaic expression and negative results. Below we indicate why we think that our new data showing this same approach causes downregulation of Cdh2 expression in the CMZ mitigates this valid concern.

1) It is important to underline that while the approach we took causes mosaic expression, Sema3fa is a <u>secreted</u> protein and so overexpressing cells can influence cells in their vicinity.
2) We previously (Halabi et al., 2022) overexpressed zebrafish Sema3fa-myc and found disruption of atrial and ventricle specific heart markers. These data argue that the zebrafish Sema3fa-myc is active.
3) Additionally, we include new overexpression data, broadening our analysis of the overexpression scenario to include Cdh2. We anticipated based on our model that Sema3f negatively regulates Cdh2 expression, that overexpression of Sema3f in the CMZ should downregulate Cdh2 expression. Indeed, this is what we observe, and now include it as data in Figure 7Q-S.

Given that we have a positive result with overexpression, which fits well with our proposed model and *sema3fa* mutant data showing more Cdh2 expression, we would like to include the overexpression data in the manuscript.

3. With regard to much of the negative data (or differences in nasal vs temporal), maternal expression could be a confound. Were maternal-zygotic embryos/larvae ever examined. This seems very doable given the non-lethal nature of the mutation.

Most of the expression data we obtained assessing the mutant CMZ showed disruption with the loss of Sema3fa, with markers of Zones 2 and 3 and progenitor proliferation impacted. Note that our analysis of *sema3fa* loss was done on embryos/larvae generated from our homozygous mutant lines, *sema3fa*$^{ca304/304}$; mutant embryos were compared to sibling embryos generated from a WT line derived from the same initial het-het cross that was used to make the mutant line. Homozygous embryos generated from a het-het cross were not used. The latter would have maternal mRNA from the oocyte. The embryos we analysed, however, would have no maternal mRNA or protein. Thus, any negative data we obtained is not because maternal mRNA or protein rescued an early developmental role for Sema3fa. We have clarified our use of *sema3fa* mutant fish in the Materials and Methods.

4. In Figure 6 – it seems that while the mutant clones are smaller, they are more numerous in number (15 clones from 14 wild-type retina; 21 clones from 9 mutant retina). This is worth discussing.

We would prefer not to comment on the numbers of clones. The retinas were serially sectioned. At 20 dpf the lens is hard and not all sections in a retina were sufficiently intact to count clones they contained; sections could rip, or we might lose the RGC layer, or sections would fold over on themselves. We restricted our analysis to those clones (clusters) where cells across all three layers could be counted across all the necessary sections. We now make this clear in the Materials and Methods.

5. In Figure 6A-B and C-D, are these different sections of the same sample/clone? If not, it looks like the clone shown in Figure 6C is from a rod progenitor, which was said to be excluded from the analysis.

These are not different sections of the same sample/clone. They are each from a different retina. We have switched the panels to more representative clone/clusters, and make sure we indicate that the examples are from different retinas. We do want to reassure the Reviewer that we did not include “clones” where only rods were present in our analysis. The choice of that “clone” was a mistake.

6. In interpreting the smaller clone sizes in Figure 6, the authors might consider the fact that RGC, the first neuron type generated, was over-represented. Perhaps this shift in neurogenic competence ‘depleted’ progenitors resulting in smaller clone sizes. Related, it is worth discussing whether the disorganization in the niche altered the probabilities of symmetric vs asymmetric type divisions within clones. This is another mechanism that determines overall clone size and composition.

We had mentioned that the RGCs were over-represented in our discussion. We now raise in the Discussion the possibility that disorganization of the niche could impact symmetric vs asymmetric type divisions that may impact clone composition and size.

7. The term “masked analysis’ instead of ‘blinded’ is the preferred.

We have made this change.

8. Lots of expression (and some other) data listed as 'data-not-shown'. This should be included as supplemental figures.

We have either included data in a new Supplemental Figure 1 or removed them from the manuscript.

Reviewer #2:

The Reviewer felt "The present study offers a potentially valuable contribution to improving our mechanistic understanding of CMZ neurogenesis.", but felt there were "shortcomings related to: the rationale of certain experiments; the specific analyses used; and the need for additional complementary evidence to further support the main conclusions". In addressing their comments below we trust we have dealt with these issues.

Major comments:

(1) The conclusions regarding changes in CMZ progenitor cell cycle are confusing and a clearer interpretation is required. In Fig 2 the authors report a reduction in PCNA 'area' in the mutant CMZ. In Fig 3, the authors report that the size and density of the mutant CMZ is normal throughout early larval stages. Together, these data would suggest that there are fewer cycling cells within the CMZ population. However, an increase in the number of EdU+ cells in the mutant CMZ is reported in Fig 2, and an increase in the 'area' of vsx2 and ccnd1 expression as a proportion of the CMZ is reported in Fig 3 and Fig 4, which would indicate an increased fraction of cells that are actively proliferating. This seems contradictory. The authors speculate in the Discussion that these data might suggest that mutant cells are stuck in S phase or G2, but this seems incongruent with the PCNA expression data (reduced) and the normal numbers of mitotic (pHH3+) cells indicating that mutant CMZ cells progress through to mitosis normally. Perhaps PCNA and EdU double labelling (or EdU cumulative labelling) could be used to more precisely discern changes in CMZ cell cycle kinetics in the mutants.

We took this very helpful suggestion of the Reviewer and performed PCNA and EdU double labeling combined with EdU cumulative labeling. The new cumulative EdU labeling indicate that at 72 hpf there are fewer total numbers of cycling cells in the CMZ of mutants as compared to WT (new Figure 2K). Analysis of the slope of the cumulative curve (pre-plateau) indicates that the S-phase and cell cycle times for CMZ progenitors in the mutants are shorter than in WT. Potentially, if cells move more quickly through G1 into S-phase (Connolly and Bogdanffy, 1993) this would be seen as both a decrease in high PCNA+ cells (those in late G1, early S-phase) (new 72 hpf data; Figure 2G'',H'',J), and a shorter cell cycle (see text). Our observation that levels of mRNA for Ccnd1 are lowered in mutants, a protein whose levels are reduced during S-phase (Stacey, 2011), also supports the idea that the G1-S transition is disrupted in mutants. The fact that progenitors that produce neurons exhibit a shorter S-phase (Arai et al., 2011) may

provide an explanation for why our retinal cohorts are smaller in size, in that progenitors may be biased towards symmetric neuronal divisions and away from non-neurogenic, expansion cell divisions that allow for expansion of the CMZ progenitor pool. We discuss this possibility in a revised Discussion.

Interestingly, our initial EdU analysis was performed at <u>7 dpf</u>, with a 4 hour pulse that the new cumulative labeling of Figure 2K indicates should be close to catching the maximal number of cycling progenitors. As the CMZ matures through larval development, the numbers of mitotic cells in the retinal periphery decrease (Hernandez-Nunez et al., 2021). At 7 dpf, Sema3fa mutants exhibit slightly more EdU positive cells (now Figure 2Q), potentially reflecting delayed maturation. The fact that, as seen at 72 hpf, fewer numbers of high PCNA+ cells are present in the mutant vs. WT CMZ (now Figure 2P), suggests that the mutant cells continue to exhibit a shortened cell cycle. Thus, an alternate explanation for the higher numbers of EdU+ cells in the mutant CMZ at 7 dpf could be that more progenitors transition through S-phase over a defined period of time; a possibility that is masked at 72 hpf with the much high numbers of proliferative progenitors in both genotypes, on top of a reduced growth fraction in mutants. Note that pHH3+ cell numbers at 7 dpf were similar between the two genotypes (Figure 2S); progenitors committed to neuron production have been shown to have a shorter M-phase (Arai et al., 2011), and thus fewer of the EdU-labeled progenitors (cumulative label) may be captured with pHH3 analysis at a single time point, resulting in comparable levels of pHH3+ cells in WT and mutants. In thinking about our data in line with what has been shown in the developing mouse cortex (Arai et al., 2011), we propose that in the mutant CMZ there is precocious commitment to be a neuron progenitor, and thus less expansion of progenitors. Over time this would result in less neuron production and a smaller retina. We consider the 7 dpf data in a revised Discussion.

(2) The data on zonal expression patterns within the CMZ is interesting and it does appear that the proportion of stem cells might be expanded in the mutant relative to progenitors. It was also interesting that CMZ cells in mutants had altered identities with misexpression of genes that normally convey a cell fate progression toward differentiation. However, the main challenge with these interpretations is the reliance on in situ expression 'area'. Using an area measurement as a proxy for the number of cells with a specific cell fate might be problematic in this context given the variation in the in situ signal, which is non-quantitative. For example, the authors show that in situ area changes in col15a1b and atoh7 positively correlate with RT-qPCR data, but in contrast the ccnd1 and cdkn1c data negatively correlate. While it is true that expression levels per cell might be different than the spatial distribution of expressing cells, the data presented do not clearly distinguish between these possibilities occurring in the mutant CMZ compared to controls. The increased overlap in the in situ expression domains of zone 2 with zone 3 genes in the mutants (there is also overlap in WT) might suggest that the markers of cell fate progression are altered but it's not clear to me how this reflects both the "phase of neurogenesis" and "location of cells" within the CMZ, as the authors concluded (lines 303-305).

We have addressed this issue of the Reviewer with new data. Our rationale for looking at the distribution of Zone markers in the mutants vs. WT was based on the idea that Sema3fa as a guidance molecule would change the localization of Sema3fa-receptor expressing cells within the CMZ. To address this possibility, we had to use an approach of investigating gene expression that allowed us to look at the spatial localization of gene expression. While RT-qPCR is valuable for examining changes in gene expression, the approach cannot address spatial aspects of mRNA expression. Thus, we needed the ISH data, which reliably, over multiple different experiments and with distinct markers, showed disorganized expression of the markers in mutants as compared to WT; hence, why expression domains were larger. To address directly whether changes in domain size correlate with the numbers of cells that express a zone marker we combined double FISH for *ccnd1* and *atoh7* with Hoescht labeling of nuclei. In this way we could identify the numbers of nuclei that sat within a domain of fluorescent ISH label for these two key genes of interest. Interestingly, we found that there appeared to be more *atoh7* expressing cells in the mutant as compared to control, which was not the case for *ccnd1*. These data are now included in new Figure 5Q,R. These data are useful because they indicate that at least one Zone 3 marker, reflecting committed progenitors, was expressed by more cells, but that the uncommitted *ccnd1* progenitors were unchanged in numbers with Sema3fa loss (though more also express *atoh7*). The latter data when combined with the larger *ccnd1* expression domain and reduced *ccnd1* mRNA levels in mutants than WT, argue that the mutant *ccnd1*+ progenitors are more dispersed and express slightly less *ccnd1*.

(3) Imaging data representing the over-expression of sema3fa-myc in Fig 2 (Fig 2R-T) is of relatively poor quality and it is difficult to discern the degree to which the myc+ transgene is expressed in EdU+ progenitor cells. It looks like most of the EdU+ cells do not express myc, perhaps due to the variegated heat-induced expression, and that might be reflected in the data showing no change in proliferation. I'm not sure this is an unambiguous test of whether transient sema3fa expression can induce changes in CMZ cell proliferation.

Note that Sema3fa is a secreted molecule. We would not expect that it would have to be expressed in a progenitor to necessarily influence the ability of that progenitor to proliferate. Instead, a Sema3fa-myc+ cell, expressing and secreting higher levels of Sema3fa than endogenous, could influence its neighbours. Indeed, in new experiments to address the concerns of all three Reviewers about the overexpression data, we performed Cdh2 immunolabeling of CMZ that overexpressed Sema3fa-myc. Based on our model, where we propose Sema3fa negatively regulates Cdh2 expression, we would expect that Sema3fa overexpression would reduce Cdh2 levels in the CMZ. Indeed, this is what we saw, and include now in a new set of panels (Figure 7Q-S). Cells neighbouring a Sema3fa-myc expressing cell showed reduced levels of Cdh2. These data argue that Sema3fa-myc is secreted and can change the behaviour of neighbouring cells. Thus, if Sema3fa promotes proliferation we would have expected some kind of proliferative response of progenitors neighbouring Sema3fa expressing cells. This was not the case. As such, we propose that Sema3fa does not directly promote CMZ proliferation. We changed the corresponding panels (now Figure 2U,V,V')

to better show that robust Sema3fa-myc overexpression within the CMZ does not increase EdU+ cell numbers.

(4) Eye size is reported to be normal in the sema3fa mutants at 72hpf as well as at 10dpf, yet significant changes to CMZ progenitor cell proliferation is reported starting as early as 72hpf. The lack of evidence for changes in eye size during the first month would suggest that overall eye size might be uncoupled from changes in CMZ neurogenesis throughout the larval period in this mutant and is contrary to the authors' main conclusion that defects in sem3fa-dependent CMZ neurogenesis is a cause of eye growth defects. Perhaps changes in eye size in mutant juvenile fish are a result of other growth processes in the eye, such as choroid and ciliary/iris growth, tissue stretching, etc., in addition to a reduction in CMZ neurogenesis. As it stands, the evidence for this correlation is not convincing. If it is a contributing factor, then perhaps showing eye size changes between 10dpf and 1mpf would help to emphasize the slow, protracted nature of this correlation making it more plausible, especially if changes are observed just after 10dpf.

We now include new data measuring eye size at 20 dpf. The Reviewer raised the issue that changes in other parts of the eye (e.g. iris), and not retinal size, might account for the differences observed in overall eye size. Thus, we measured the circumferential length of the inner plexiform layer (IPL), as a measure internal to the neural retina, in retinal sections of 20 dpf fish. The IPL in mutants is significantly smaller than WT. These data are included in the text of the Results.

(5) The pulse chase experiments reported in Fig 7 indicate that in the mutant, fewer new cells are added to the retina from the CMZ. The authors test the hypothesis that sema3fa is a positive regulator of repulsion, or negative regulator of cell adhesion, within the CMZ leading to mutant cells being less motile. To do this, they first induce the over-expression of sema3fa and look for proximity of transgene (myc+) expressing cells with neighboring cells expressing sema3fa receptor Plxna1. The rationale for this experiment is unclear. Plxna1 receptors appear to be expressed in all the CMZ cells (Fig 1L, O), so it is not surprising that there are no differences in proximity. Is there an increase in the level of plxna1 receptor expression rather than an increase in area of expression? Can the authors validate this with RT-qPCR or other methods? Also, the control used in this experiment is constitutive GFP expression rather than a heat-inducible construct like the sema3fa-myc. It would be more appropriate to use a heat inducible control.

The new data showing reduced expression of Cdh2 with Sema3fa overexpression do provide support that the Sema3fa overexpression system worked. Our original idea was that CMZ progenitors that express Plxna1 would move away from a high source of Sema3fa. We had seen this as solely a repulsion mechanism, and not changes in Plxna1 receptor expression. We have removed the Plxna1 data, given problems with interpretation of the data based on the Reviewer's point that many CMZ cells express Plxna1, and that we now have data suggesting regulation of adhesion as a mechanism to explain the defects seen in the *sema3fa* mutant CMZ.

(6) Perhaps a better test of a potential sema3fa repulsion mechanism for CMZ cells would be to induce the over-expression and then perform an EdU pulse-chase experiment to see if cohorts of labeled cells show increased motility as they emerge from the CMZ. The alternative explanation suggested by the authors was that mutant CMZ cells have increased adhesion. If so, then the same sema3fa over-expression experiment should result in a decrease in expression levels of cell adhesion markers. Was this examined?

We tried the experiment as suggested by the Reviewer, but struggled with how to interpret the data, and so will not include the data in a revised manuscript. The heat shock results in transient Sema3fa overexpression. Thus, while we could see cohorts of EdU+ cells that had emerged from the CMZ at 7 days post-heat shock, we had no way of correlating these data to embryos where we had successful expression in the CMZ of the Sema3fa-myc construct. Further, in these experiments the overexpression is not targeted only to the CMZ but also to cells of the mature retina. If indeed, Sema3fa acts as a repellent, we would have a repellent being overexpressed both in the CMZ, but also where the CMZ progeny were migrating – since these cells express Nrp2b, they should continue to be responsive to Sema3fa. Thus, how the emerging progeny would behave is not easy to predict. The new data showing reduced expression of Cdh2 with Sema3fa overexpression, however, do provide additional support, alongside the upregulation of Cdh2 in the *sema3fa-/-* CMZ, for our model that Sema3fa regulates cell adhesion molecule expression.

As suggested by the Reviewer, we include new data showing that Sema3fa overexpression decreases expression of a cell adhesion molecule, Cdh2 (Figure 7Q-S).

(7) The authors note that there are no changes in cell death in the embryonic sema3fa mutant retina. However, it is possible that one reason for the reduced size of the EdU+ cohorts in the pulse-chase experiments is due to increased cell death of newborn cells in the larval mutant retina. The authors should check for this possibility using an apoptotic marker.

We now include TUNEL data for *sema3fa* mutant retinas at 7 dpf when CMZ neurogenesis is occurring in the text of the Results. Importantly, little cell death occurs in the normal larval retina, and there is no increase in cell death observed for *sema3fa* mutants over WT. Thus, newborn cells arising from the CMZ in both WT and mutant eyes, which would have been happening for at least 5 days (from 2-7 dpf) at the time of our analysis, do not appear to die at any noticeable rate in the *sema3fa* mutants.

(8) The data represented in the graphs in Fig 6J-M is confusing. Is standard deviation being shown on the y-axes? The data are analyzed by MW statistics which accounts for ranks between the data points. Are the authors performing a test of significance on the standard deviation of the data? This is not clearly explained, but if I understand the goal of this analysis correctly, I recommend that the authors use a non-parametric ANOVA to analyze this data for the type of inferences they are trying to make.

We apologize for not being clear. Yes, the goal of the analysis was to show that the distribution of cells in mutant cohorts was more variable than in WT, and so we graphed the standard deviation for the cell distribution present in cohorts of a single retina. We have rewritten the description of this analysis in the text of the Results and legend for Figure 6 to be more clear, and used a Levene's test of the equality of variance to statistically compare the data.

Minor comments:

(1) The expression of sema3fa at 1mpf appears different using the DNP probe (Fig 1S) vs the DIG probe (Fig 1V). This might just be the choice of representative images, but the sema3fa-DIG expression more closely overlaps with postmitotic/differentiating progenitors rather than the actively proliferating progenitors. Can the authors comment?

We now outline the CMZ in the Figures so that it is clearer in the CMZ where *sema3fa* is localized. With both probes we find expression in the CMZ where actively proliferating prognitors reside. We directly address this point and show co-localization with *ccnd1* in new Supplementary Figure 1A,B. Note that Figure 1S-U is a more peripheral retinal section than that shown in Figure 1V-X, so the anatomy of the CMZ differs slightly. In general, we find our DIG probes work better than our DNP probes and so this changes the intensity of the label, but not the localization.

(2) The Crb2a and aPKC expression data in Fig 4 exemplify the polarized nature of the of the CMZ neuroepithelial cells and are not specific stem cell markers as suggested by the authors. These apical markers are expressed throughout the CMZ and ONL in embryonic and early larval retina.

We have altered text to not suggest these are specific stem cell markers, though we find that within the CMZ both markers are more highly expressed in the distal CMZ containing the retinal stem cells.

(3) For the EdU pulse-chase experiments described in Fig 6 and Fig 7, the authors refer to "clones" of EdU+ cells. Strictly speaking, the analysis is not a clonal analysis of lineages derived from single-labeled CMZ cells. Instead, it is a "cohort" of progenitors that are initially labeled with the EdU pulse, and these cells (and their progeny) are followed over the next two weeks. I recommend the authors clarify this description for accuracy. Also, in Fig 6A-D, the image panels are referred to as "14 day pulse" but it should read "14 day chase".

We changed clone to cohort, and appreciate the suggestion by the Reviewer. We have changed "14 day pulse" to "14 day chase".

Reviewer #3:

We appreciate that the Reviewer liked our "nice study", felt that it was a "carefully

designed and executed study presenting interesting results that generally support the conclusions", and that it made "a significant advance in our understanding of spatial patterning of the retinal CMZ". They suggested several points that they felt we should consider to improve the paper.

1) The authors use in situ hybridization to study expression of Sem3fa and its receptors in the CMZ at 72hpf and 7dpf in Figure 1. While the general expression pattern does appear to support the authors' conclusion that Sema3fa is expressed in CMZ progenitors, while the receptors are expressed in cells closer to differentiated central retina, co-labelling with CMZ markers would help make this point more solid. Even though cyclinD1 was used to identify proliferating cells, markers more specific for zones 1, 2 and 3 would be useful here. For example, the authors suggest that lack of labelling for Sema3fa in the most distal part of the CMZ suggest that stem cells do not express Sema3fa, but positive labeling of this domain would be more convincing to suppor this conclusion. Also, there are many mentions of "data not shown" in this section, which is now unusual given that space is not an issue. I would suggest showing this data.

We now include co-expression data for *ccnd1* and *sema3fa*, illustrating the overlap of *sema3fa* mRNA with the Zone 2 marker *ccnd1,* that labels actively proliferating progenitors. We include these data and the "data not shown" receptor data of the original manuscript in a new Supplemental Figure 1.

2) The authors may want to consider presenting the data on eye size (Figure 2) after showing disruption of spatial patterning in the CMZ of Sema3fa mutants (Figure 3). I feel like it would be a nicer flow to show what happens in the CMZ in absence of Sema3fa before showing the resulting outcome of this disorganization. Just a suggestion.

We had initially written the manuscript with the order suggested by the Reviewer. In presenting the work in oral format, we realized we prefer the order where we use the change in eye size to provide the rationale for our further analysis of the mutant CMZ. As such, while we appreciate the Reviewer's suggestion and understand completely their point, we have decided to retain the organization as per the original submission

3) The authors studied the impact of Sema3fa inactivation on the regulation of eye size, which is an important function of the CMZ. But another important function of this stem cell niche is the regenerative response after injury. I was wondering if this is also affected in Sema3fa. If not, it would suggest that different mechanisms are at play, which would be quite interesting. If the authors have data on this or can perform the experiment easily, it seems to me like an important question to address. If not, then discussion of this point may be sufficient.

This is an excellent question and one we are pursuing in a project of a PhD student in the lab. The regeneration work, and its mechanistic understanding, is a study in and of its self. Thus, we have restricted our handling of this subject to a suggestion in the Discussion.

4) In Figure 2 and 7, the authors use a myc-tagged version of Sema3fa to determine whether it acts directly on progenitors to stimulate proliferation (Fig. 2) or as a repulsive cue (Fig. 7). In both experiments, the authors fail to report any changes by the expression of the Sem3fa-myc, arguing against a direct role in proliferation or repulsion. However, these are negative results and a positive control to show that the Sema3fa-myc is functional and can elicit an expected response is missing. This is important to add given that the results obtained are negative with this tool.

This was a concern raised by all three Reviewers. As addressed in our response to Reviewer 1 we now provide positive data revealing a change in Cdh2 expression in our Sema3fa-myc overexpression experiments. These data are now included in Figure 7Q-S.
